# An artificial intelligence algorithm for automated blastocyst morphometric parameters demonstrates a positive association with implantation potential

**DOI:** 10.1038/s41598-023-40923-x

**Published:** 2023-09-05

**Authors:** Yael Fruchter-Goldmeier, Ben Kantor, Assaf Ben-Meir, Tamar Wainstock, Itay Erlich, Eliahu Levitas, Yoel Shufaro, Onit Sapir, Iris Har-Vardi

**Affiliations:** 1 The Medical School for International Health and the Faculty of Health Sciences, Ben-Gurion University of the Negev, Beer-Sheva, Israel; 2 Fairtility Ltd., Tel Aviv, Israel; 3 Fertility and IVF Unit, Department of Obstetrics and Gynecology, Hadassah Medical Organization and Faculty of Medicine, Hebrew University of Jerusalem, Jerusalem, Israel; 4 School of Public Health, Faculty of Health Sciences, Ben-Gurion University of the Negev, Beer-Sheva, Israel; 5 Fertility and IVF Unit, Department of Obstetrics and Gynecology, Soroka University Medical Center, Beer-Sheva, Israel; 6 Infertility and IVF Unit, Beilinson Women’s Hospital, Rabin Medical Center, Petach-Tikva, Israel; 7 The Sackler Faculty of Medicine, Tel Aviv University, Tel Aviv, Israel

**Keywords:** Medical research, Embryology

## Abstract

Blastocyst selection is primarily based on morphological scoring systems and morphokinetic data. These methods involve subjective grading and time-consuming techniques. Artificial intelligence allows for objective and quick blastocyst selection. In this study, 608 blastocysts were selected for transfer using morphokinetics and Gardner criteria. Retrospectively, morphometric parameters of blastocyst size, inner cell mass (ICM) size, ICM-to-blastocyst size ratio, and ICM shape were automatically measured by a semantic segmentation neural network model. The model was trained on 1506 videos with 102 videos for validation with no overlap between the ICM and trophectoderm models. Univariable logistic analysis found blastocyst size and ICM-to-blastocyst size ratio to be significantly associated with implantation potential. Multivariable regression analysis, adjusted for woman age, found blastocyst size to be significantly associated with implantation potential. The odds of implantation increased by 1.74 for embryos with a blastocyst size greater than the mean (147 ± 19.1 μm). The performance of the algorithm was represented by an area under the curve of 0.70 (p < 0.01). In conclusion, this study supports the association of a large blastocyst size with higher implantation potential and suggests that automatically measured blastocyst morphometrics can be used as a precise, consistent, and time-saving tool for improving blastocyst selection.

## Introduction

Since the birth of Louise Brown, the first baby to successfully be born from in-vitro fertilization (IVF) methods, the pregnancy rate of women treated with IVF has steadily increased. In the United States, implantation rates have increased from 27.6% in 2003 to 41.6% in 2020 in women younger than 35 years of age for non-preimplantation genetic testing (PGT) fresh embryo transfers from non-donor oocytes^1,2^. The optimization of embryo culture conditions has contributed to this increase in implantation rate. Optimization of culture conditions includes extended embryo culture for up to six days, to the blastocyst stage^3–6^. Delaying embryo transfer to the blastocyst stage seems to improve uterine and embryonic synchronicity resulting in greater live birth rates^7,8^. Gardner and Schoolcraft developed a blastocyst grading system that focuses on blastocyst expansion level and trophectoderm (TE) and inner cell mass (ICM) integrity to aid in the selection of a high-quality blastocyst^9^.

Previous publications show that blastocysts with a better expansion grade have greater implantation, pregnancy, and live birth rates following transfer^10–17^. Other investigators have found a positive correlation between blastocyst diameter, width, and area to clinical pregnancy rate^18,19^. TE quality has also been shown to be associated with implantation rate and live birth rate^13,17^. Some of these studies have found ICM grade did not predict implantation potential nor live birth rate^13,14,17^. However, other studies have found an association between ICM morphometric measurements and implantation potential. When analysing expanded blastocysts, studies have demonstrated a strong relationship between the size and shape of the ICM to implantation potential^15,20^. Research by Almagor and colleagues found that embryos with a high ICM-to-blastocyst diameter ratio had significantly increased pregnancy rates in SETs^21^.

An important technological advance in the field of assisted reproductive technology (ART) is the time-lapse monitoring (TLM) system, which was created to enable continuous embryo monitoring without removal from the incubator for frequent observations of embryonic development^22,23^. The data obtained from TLM provide raw images and videos rich with information that can be used in artificial intelligence (AI) technology to aid in embryo selection. This information is used in AI models that have been developed to annotate morphokinetic events^24,25^, detect blastocyst morphology^26–29^, and identify embryos with greater blastocyst quality^30^. Other algorithms have been created to predict clinical outcome such as clinical pregnancy^27,28,31,32^ and implantation potential^19,29^. Although the aforementioned studies show great promise, there are debates in the scientific literature as to TLM’s applicability between clinics, predictive value, and contribution to IVF clinical outcome^33–37^.

The incorporation of TLM data into AI technologies has introduced the concept of automatization of embryo selection. An advantage of automated embryo selection is the removal of subjectivity. Subjective embryologist annotation of blastocyst morphology grading may result in inconsistent findings between labs due to intra- and inter-observer variability^38,39^. Therefore, an objective automated analysis of embryos is important for reliability^40,41^. One such objective tool is a deep learning algorithm based on artificial neural networks (ANNs), such as convolutional neural networks (CNNs), that automatically analyse embryos. Several AI models have already been created to automatically define blastocyst morphology^26^, grade blastocysts^30,42^, and annotate morphokinetics^24^. Furthermore, Tran et al. developed a deep learning algorithm that could directly analyse the entire raw time-lapse video without the need for annotated parameters, making use of every data point collected from TLM to predict the probability of clinical pregnancy^31^.

The purpose of the present study was threefold: (1) to present a novel approach in blastocyst analysis that uses automatic measurements by an AI tool; (2) to study the association between automatically measured blastocyst morphometric parameters and implantation rate; and (3) to demonstrate the predictive power of a newly developed algorithm on implantation rate for its use as a future tool in embryo selection.

## Results

Data from 608 day-5 transferred blastocysts was analysed. Two hundred (32.9%) of the transferred embryos had a positive known implantation data (KIDp) and 408 (67.1%) of the transferred embryos had a negative KID (KIDn). The overall mean age of patients in this study was 33.5 years (19–45 years) with KIDp embryos associated with a younger maternal age compared with KIDn embryos (30.9 years and 34.8 years, respectively; p < 0.001, Table 1).

**Table 1:** Morphometric blastocyst parameters of embryos with known implantation data (KID).

Parameter	KIDp (*N* = 200)	KIDn (*N* = 408)	P-value
	Mean ± SD	Mean ± SD	
Woman age (years)	30.9 ± 5.3	34.8 ± 6.3	< 0.001
Blastocyst size (μm)	152 ± 19.2	144 ± 18.5	< 0.001
ICM size (μm)	76.8 ± 12.0	77.0 ± 12.8	0.898
ICM-to-blastocyst size ratio	0.507 ± 0.090	0.536 ± 0.092	< 0.001
ICM shape	1.43 ± 0.344	1.40 ± 0.298	0.313

*KIDp* known implantation data positive, *KIDn* known implantation data negative, *SD* standard deviation, *ICM* inner cell mass.

Analysis of the automated blastocyst morphometric measurements was performed at the mean time to blastocyst expansion minus the mean time to pronuclear fading (tEB-tPNf; 85.98 ± 5.18 h). The analysis demonstrated that KIDp embryos had significantly larger blastocyst sizes compared to the blastocyst sizes of KIDn embryos (152 ± 19.2 µm and 144 ± 18.5 µm, respectively; p < 0.001, Table 1). However, no significant differences were found between KIDp and KIDn embryos (Table 1) regarding ICM size (76.8 ± 12.0 µm and 77.0 ± 12.8 µm, respectively; p = 0.898) and shape (1.43 ± 0.344 and 1.40 ± 0.298, respectively; p = 0.313). Embryos that resulted in implantation had a smaller ICM-to-blastocyst size ratio than did embryos that did not result in implantation (0.507 ± 0.090 and 0.536 ± 0.092, respectively; p < 0.001, Table 1). This finding stems from a significant difference in blastocyst size between implanted and non-implanted embryos and not from a difference in ICM size. Therefore, it seems that implanted embryos included more expanded blastocysts than did non-implanted embryos.

A multivariable logistic regression analysis was performed. Although ICM size was not found to be significant in the univariable logistic regression analysis, it was included in the multivariable logistic regression analysis due to its clinical importance. Blastocyst size had a significant positive association with implantation such that with every 1 μm increase in blastocyst size, there was a relative increase in the odds of implantation by 2.1% (adjusted OR 1.02, 95% CI 1.01–1.03; p < 0.001, Table 2). Woman age had a significant negative association with the odds of implantation (adjusted OR 0.898, 95% CI 0.870–0.926; p < 0.001, Table 2). In another multivariable logistic regression analysis, blastocyst size was replaced with ICM-to-blastocyst size ratio. ICM-to-blastocyst size ratio was significant in this multivariable analysis despite including the nonsignificant variable of ICM size. Since ICM size does not significantly differ between implanted and nonimplanted embryos, it is the component of blastocyst size in the variable of ICM-to-blastocyst size ratio that makes this parameter a significant predictor of implantation potential.

**Table 2:** Multivariable logistic regression analysis assessing the independent effect of ICM size, blastocyst size, and woman age on implantation potential.

Variable	Adjusted OR (95% CI)	P-value
ICM size	0.987 (0.973–1.002)	0.083
Blastocyst size	1.02 (1.01–1.03)	< 0.001
Woman age	0.898 (0.870–0.926)	< 0.001

*OR* odds ratio, *CI* confidence interval, *ICM* inner cell mass.

Based on the results of the multivariable logistic regression analyses, embryos were divided into two groups according to blastocyst sizes larger than the mean size (147 ± 19.1 μm) (group 1) and blastocyst sizes smaller than the mean size (group 2). A significantly higher rate of implantation was found in group 1 as compared to group 2 (41.2% vs. 25.8%, respectively; OR 2.01, 95% CI 1.43–2.84; p < 0.001). The independent effect of this criterion on implantation potential was analysed and adjusted for woman age. Among women whose embryos met the criterion for inclusion in group 1, the odds for implantation increased by 1.74 as compared to embryos from women that did not meet the criterion (95% CI 1.22–2.50; p = 0.002, Table 3). As previously demonstrated, woman age maintained its negative association with implantation potential (adjusted OR 0.898, 95% CI 0.871–0.927; p < 0.001, Table 3). The performance of the algorithm, which included woman age and analysed embryos according to the aforementioned criterion, is represented by an area under the curve (AUC) of 0.70 (SE = 0.02, 95% CI 0.653–0.738, p < 0.01). The performance of a model analysing woman age alone is represented by an AUC of 0.68 (SE = 0.02, 95% CI 0.640–0.726, p < 0.01). The difference between the aforementioned AUCs was not significant (p = 1).

**Table 3:** Multivariable logistic regression analysis, adjusted for woman age, assessing the independent effect of blastocyst size on implantation potential.

Variable	Adjusted OR (95% CI)	P-value
Blastocyst size > mean	1.74 (1.22–2.50)	0.001
Woman age	0.898 (0.871–0.927)	< 0.001

*OR* odds ratio, *CI* confidence interval.

## Discussion

The most significant contribution of the current publication is the addition of automated measurements of blastocyst morphometrics to the embryo selection process. To the best of our knowledge, this is the first study to show a correlation between automatically measured blastocyst morphometric parameters and implantation. Morphometrics that are determined automatically by ANNs reduce intra- and inter-observer variation between embryologists by providing consistent and objective measurements, and save time spent on manual measurements. In addition, the algorithm developed in this study predicted increased implantation rates among patients whose embryos had a blastocyst size larger than the mean with an AUC of 0.70 (SE = 0.02, 95% CI 0.653–0.738, p < 0.01). This AUC of 0.70 is greater than the AUC of an algorithm analysing implantation rates using woman age alone (AUC 0.68, SE = 0.02, 95% CI 0.640–0.726, p < 0.01). Furthermore, the difference between these AUCs was not significant (p = 1).

The results from the present study demonstrate that blastocyst size and woman age are independently associated with implantation potential. These results support findings in publications that found a positive correlation between blastocyst expansion degree and implantation rate^15,16,18,19^ and a negative correlation between woman age and implantation^43–46^.

In the current study, the morphometric parameters of ICM size and ICM shape were found to be not significantly associated with implantation potential. In contrast, Richter et al. found that in expanded blastocysts, implantation was increased in women whose blastocysts had a large ICM area and/or slightly oval ICM shape^20^. The same study showed that blastocyst size was not significantly associated with implantation. The discrepancies between Richter et al. and the present study may be explained by differing observation methods and culture conditions. Richter et al. examined the embryos at 24-h intervals and, therefore, it is possible that certain key developmental changes were missed. Furthermore, the method of observation employed by Richter et al. did not allow for continuous and undisrupted culture conditions, which is known to negatively affect embryo development and quality^47,48^ and would thus affect the results of the study. In addition, Richter and colleagues included the zona pellucida in their measurement of blastocyst size, a method which was not employed in this study as the zona pellucida undergoes considerable change throughout blastocyst expansion leading to inconsistent measurements. In this study, blastocyst size was calculated by a bounding box around the outer part of the TE cells, excluding the area occupied by the zona pellucida, and defined as the average between the width and height of the outer part of the TE.

Like the present study, Almagor and colleagues analysed the relationship between the ICM and blastocyst diameter relative to implantation. They demonstrated that in pre-expanded blastocysts, implanted embryos had significantly larger ICM-to-blastocyst diameter ratio compared to non-implanted embryos^21^. The present study found the opposite when analysing the ratio in expanded blastocysts: ICM-to-blastocyst size ratio was smaller in implanted embryos than in non-implanted embryos. It is possible that the relative size of the ICM-to-blastocyst holds different importance at different stages of development and would explain the variations between the present study and Almagor’s study. Furthermore, this study utilized automatization of blastocyst size measurement while Almagor et al. and Richter et al. utilized manual measurements.

The use of AI algorithms to predict clinical outcome in ART has already shown great promise^19,27–29,31,32^. Bori et al. incorporated manually measured blastocyst morphometrics and morphokinetics in an ANN to predict implantation with relatively good performance^19^. Although the present study did not include morphokinetics in its algorithm, it introduces the novelty of automated morphometric measurements. Additionally, Bori and colleagues included only embryos from oocyte donations while the present study analysed embryos from autologous oocytes. Analysis of embryos solely derived from oocyte donation limits Bori’s algorithm to the prediction of high-quality embryos derived from young patients with likely high-quality oocytes. The analysis of embryos originating from autologous oocytes and from women of a wide range of ages makes the algorithm of the present study more widely applicable.

One strategy employed by AI models is the analysis of blastocyst morphology at an endpoint. Diakiw et al. developed an AI model that involved the deep learning analysis of the embryo on day 5 of culture. AI scores were created to represent the likelihood of clinical pregnancy and thus provided a qualitative score for blastocyst selection. The AI scores of the study were significantly correlated with known morphological features of embryo quality based on the Gardner criteria^28^. Chavez-Badiola et al. created an AI model dubbed ERICA that analysed embryos on day 5 or 6 of culture and provided a qualitative ranking system for the prediction of embryo ploidy and implantation^29^. Similarly, our study developed an AI model to analyse the blastocyst on day 5 of culture, but, at a specific blastocyst developmental stage (mean tEB-tPNf). This time was chosen for blastocyst morphometric measurement as it is the time of development when the ICM and TE borders are clearly seen and can therefore be most accurately measured. Before the time of blastocyst expansion, the embryo has not significantly increased in size and therefore, it would be difficult to find differences in blastocyst sizes between embryos. This novel timepoint for morphometric measurement presents a new viewpoint into the blastocyst selection process, which this study has shown is associated with the likelihood of implantation and has not yet been described in the automatization of AI.

Although the present study includes woman age, an important confounding factor in ART, it lacks some information on patient characteristics, such as BMI, number of oocytes retrieved, and semen analysis, which could be additional confounders. Another limitation includes the retrospective nature of the study and that automatic morphometric measurements were performed on day-5 blastocysts that were preselected based on morphokinetic parameters and Gardner criteria, which may introduce a selection bias.

Despite the limitations, the study has several notable strengths. First and foremost, this is the first study of its kind to date to automatically measure blastocyst morphometric parameters without time-consuming manual annotation. The study population included embryos collected from three different IVF centres each using the same time-lapse system and culture conditions. Therefore, the results of the present study can be more widely applied to other centres using the same conditions. Additionally, women from a wide range of ages were included in this study. This reflects clinical practice as centres often see patients from a variety of ages and thus further increases the relevance of the study’s algorithm. Furthermore, our study provides a quantifiable measurement for the likelihood of implantation, which can be applied for practical clinical use. Although our automatic morphometric method was performed on a group of preselected embryos, we demonstrated that even in such a group, the odds of implantation are improved. In other words, all else equal, embryos with blastocyst sizes greater than the mean had an almost two-fold greater odds of implantation than did embryos with blastocyst sizes smaller than the mean (adjusted OR 1.74, 95% CI 1.22–2.50; p = 0.002, Table 3).

In conclusion, the selection of embryos using an algorithm based on automatically measured blastocyst size may serve to improve clinical outcome relating to increased implantation potential. The comprehensive automatization of blastocyst parameters should increase the consistency and accuracy of blastocyst measurements. Furthermore, automatization should decrease the amount of time embryologists spend on blastocyst measurements and increase the predictive power of the algorithm for improvement of clinical outcome. Future research will include the application of this algorithm to prospective embryo selection. In addition, the algorithm will incorporate automatically measured blastocyst morphokinetics following research demonstrating the reliability of these measurements.

## Materials and methods

A retrospective nested case–control study was conducted and included 608 embryos from women who underwent IVF treatment in three public IVF units between 2014 and 2017. The protocol was approved by the Soroka University Medical Center Institutional Review Board (IRB number: 0006-20HMO). All experiments were performed in accordance with the relevant guidelines and regulations. Due to the retrospective nature of the study, the review board of Soroka University Medical Center approved the study with deidentified data and without requiring individual informed consent from each patient.

The inclusion criteria were: (1) Patients who underwent an IVF procedure of day-5 blastocyst transfers with continued growth monitoring via Embryoscope with known implantation data (KID). (2) The number of the transferred embryos was one or more. (3) Transferred embryos resulting in no implantation (KID negative, KIDn) or in which the number of gestational sacs with foetal heartbeat matched the number of transferred blastocysts (KID positive, KIDp). The exclusion criteria were: (1) Frozen embryo transfer cycles (the morphological appearance of the thawed blastocyst may differ from its fresh state since during the freezing process, the blastocyst undergoes collapse and not all thawed blastocysts return to their original expansion). (2) Transfers of embryos that underwent preimplantation genetic testing (PGT). (3) Embryos from donor oocytes.

### Ovarian stimulation and luteal support

In this study, two ovarian stimulation protocols were used: the gonadotropin-releasing hormone antagonist and the long gonadotropin-releasing hormone agonist protocols in combination with either human menopausal gonadotropin or recombinant follicle stimulating hormone.

### Oocyte retrieval and fertilization

Cumulus-oocyte complexes were cultured in fertilization medium (Life Global®, Cooper Surgical, Brussels, Belgium) at 37 °C, 5.7% CO_2_, and 5% O_2_. Fertilization was performed by insemination or by intracytoplasmic sperm injection (ICSI). Before ICSI, oocyte denudation was initiated by incubation in 80 IU/mL of hyaluronidase (Irvine Scientific, Santa Ana, CA, USA) followed by mechanical pipetting to remove the cumulus cells from the oocyte. ICSI procedures were performed using a Nikon Eclipse Ti microscope. The inseminated oocytes were inserted into the slides one day after oocyte retrieval when mechanical denudation from cumulus and corona radiata cells was completed.

### Embryo culture and imaging system

Immediately after the ICSI procedure, or the day after insemination and oocyte denudation, the oocytes were placed in culture slides (EmbryoSlide, Vitrolife A/S, Aarhus, Denmark) containing 12 micro-wells, each filled with 25-µL droplets of a single step Global medium or one-step medium “SAGE 1step” (SAGE, Al-rad medical, Nes Ziona, Israel), and covered with mineral oil. The slides were prepared 17 h in advance and left in an incubator to pre-equilibrate at 37 °C in 5.7% CO_2_. The oocytes were incubated in a time-lapse incubator—an EmbryoScope™ system at 37 °C, 5.7% CO_2_, and 5% O_2_ (Vitrolife A/S, Aarhus, Denmark). Images were acquired at intervals of 15 min through several focal planes and obtained data was evaluated on an Embryo Viewer® workstation external computer (Vitrolife A/S, Aarhus, Denmark). All focal planes were used for annotation and three central focal planes (− 15, 0, 15) were used as model input.

### Embryo selection and transfer

This study included data analysis of videos obtained from embryos cultured in time-lapse incubators (Embryoscope, Vitrolife). The clinical data collected from the patients’ medical files included age, fertilization method, and number of transferred and implanted blastocysts. Before embryo transfer, annotation of morphokinetic events during embryonic development and blastocyst grading were performed by one well trained embryologist using Embryo Viewer®. Oocyte fertilization was confirmed by the presence of two pronuclei (2PN). All the relevant morphokinetic events included time of pronuclear fading (tPNf) to time of expanded blastocyst (tEB). tPNf was calculated as time 0 to enable a similar starting point for the annotation of blastocysts originating from insemination or ICSI procedures. tEB was defined as the time to form a full blastocyst, consisting of an expanded blastocoel cavity and well-defined ICM and TE^49^. Embryos were cultured for five days and transferred at the blastocyst stage. Blastocyst selection was based on morphokinetic parameters and Gardner criteria^9^. Blastocyst culture and transfer were based on patient and physician decision. Embryos were transferred using an abdominal ultrasound-guided technique.

### Blastocyst measurements

**Figure 1:**
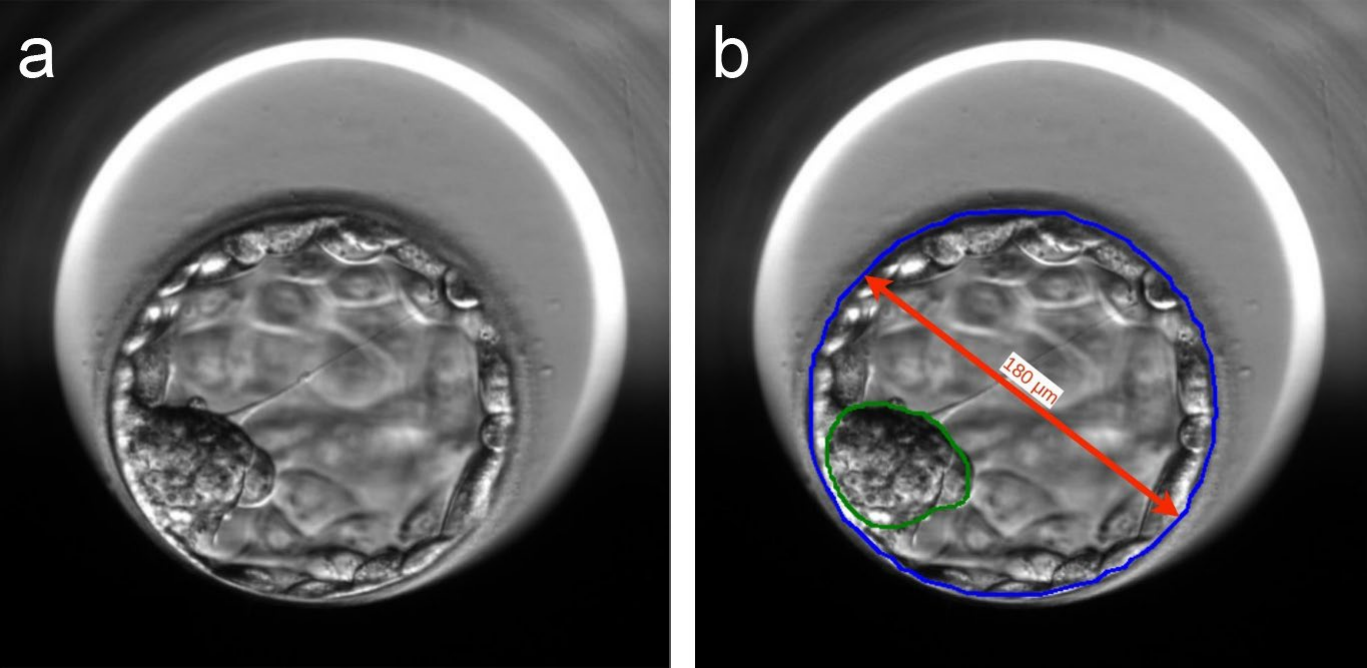
Automatic measurements of blastocyst morphometrics. (**a**) A day-5 blastocyst with trophectoderm (TE) and inner cell mass (ICM) cells. (**b**) The same embryo with markings. Blue represents the hand drawn segmentation around the outer part of the TE cells, excluding the area occupied by the zona pellucida. Green represents the ellipse that best fits around the segmented ICM pixels in the least-squares sense. Red represents the diameter of the embryo.

A retrospective quantitative measurement of blastocyst morphometric parameters of all the 608 transferred blastocysts with KID were performed. To measure the blastocyst morphometric parameters at the same time of development for all the transferred blastocysts, the morphometric variables were measured at the mean time of blastocyst expansion minus the mean tPNf (tEB-tPNf; 85.98 ± 5.18 h). For each blastocyst, the following measurements were performed: blastocyst size (μm), ICM size (μm), ICM-to-blastocyst size ratio, and ICM shape. The model outputs a pixel mask with a value of 1 for each pixel belonging to the embryo while excluding the zona pellucida (i.e., the outer part of the TE). The model was trained to match the hand drawn segmentation of an expert embryologist (Fig. 1; see “Training of the segmentation models”). From this raw pixel output, the blastocyst size was calculated by finding a bounding box around all the pixels belonging to the embryo followed by taking the average of the width and height of the bounding box. This value was defined as the blastocyst size. To determine the ICM size of the blastocyst, the ICM model first outputs a pixel mask that corresponds to the hand drawn segmentation (Fig. 1; see “Training of the segmentation models”). Then, an ellipse that best fits around the segmented ICM pixels in the least-square sense was determined (implemented in the OpenCV library using function cv.fitEllipse()). To get a single number to correspond to the ICM size, ICM size was defined as the long diameter of the fitted ellipse. The ICM-to-blastocyst size ratio was defined as the ICM size divided by the blastocyst size (with ICM size and blastocyst size calculated as previously described). ICM shape was calculated as the long diameter of the fitted ellipse divided by the short diameter. In cases of ICM shape = 1, the shape is round and in cases of ICM shape > 1, the shape is more elongated.

### Training of the segmentation models

Expert embryologists manually marked tight polygons around the ICM and outer parts of the TE. The semantic segmentation neural network model was trained on these hand drawn segmentations and output a pixel-level annotation for each object. The number of videos and frames annotated for each task is shown in Supplementary Table 1. The frames do not include cleavage stage embryos and include embryos from the start of blastocyst (tSB) until the last frame before embryo transfer. All segmentation models are based on the AI technology known as CHLOE^TM^ by Fairtility LTD which used Mask-RCNN neural network architecture with a ResNet50 backbone and with Feature Pyramid Networks. The model was pretrained on the ImageNet and MS-COCO datasets. As for image preprocessing, for the TE model, the images were resized to a 384 × 384-pixel resolution. For the ICM model, the images were cropped to contain only the embryo (using the TE model) and then resized to a 224 × 224-pixel resolution. Train/validation/test were determined by randomly splitting the annotated videos between the three sets, and then taking the frames. This ensures that frames from a single video were not split between train/validation/test. For the TE model, one video was kept for model validation and 32 videos were kept for model testing (Supplementary Table 1). One video for validation was enough to choose a model that demonstrated very high accuracy (nearly 100%) on the test data, so we opted to keep all the rest as training data.

For each frame, the overlap between the model-predicted pixel mask and the embryologist-annotated pixel mask was calculated using Intersection Over Union (IOU). Predicted mask was treated as a correct prediction if the overlap between the predicted mask and the expert annotated mask crossed a certain threshold. The number of frames in which the model-predicted TE/ICM overlapped with embryologist-annotated TE/ICM in more than 50% of the pixels was 99.9% and 95.9%, respectively. Furthermore, the number of frames in which the model-predicted TE/ICM overlapped with the embryologist-annotated TE/ICM in more than 75% of the pixels was 99.9% and 72.8%, respectively.

### Outcome measures

Embryo implantation was confirmed by the presence of a gestational sac with a foetal heartbeat by a transvaginal ultrasound examination six weeks following oocyte retrieval.

### Data analysis and statistical methods

Statistical analysis was performed with SPSS statistical software version 29^th^ edition (SPSS Inc., Chicago, IL). The studied embryos were divided into groups (cases and controls), for each of the studied outcomes. The χ^2^ test was used to compare categorical variables. Data on continuous variables, including all blastocyst morphometric values, were expressed as mean ± standard deviation and compared using two-sided Student’s t-test with an alpha of 0.05. All continuous variables were tested a-priori for normal distribution, using a histogram curve, mean and standard deviation, skewness and kurtosis. Logistic regression analysis was used to identify the independent association between the different time intervals and/or the different morphometric parameters and KID results while adjusting for maternal age. The correlation between all the covariables was checked and only those without a significant correlation to one another (r < 0.6, p < 0.05) were included in the multivariable regression analyses. The adjusted odds ratio (OR) and 95% confidence interval (95% CI) were computed. A p-value < 0.05 was considered significant.

The performance of the algorithm was assessed using receiver operating characteristics (ROCs). The ROC curve is depicted by plotting the true positive rate (TPR) against the false positive rate (FPR) at various thresholds. The accuracy is measured by the area under the ROC curve (AUC). AUC may be used to represent the discriminative performance of a binary classifier and thus was the most appropriate measure for the study's model to identify blastocysts according to odds of implantation. ROC characteristic analysis was conducted based on several logistic models, and the corresponding AUC was compared between them using the DeLong method.

### Supplementary Information

Supplementary Table 1.

## Data Availability

The datasets generated and/or analysed during the current study are not publicly available as they are property of Fairtility LTD but are available from the corresponding author upon reasonable request.
