# Supplementary material for: An artificial intelligence algorithm for automated blastocyst morphometric parameters demonstrates a positive association with implantation potential
Source: Sci Rep. 2023 Sep 5;13:14617. doi: 10.1038/s41598-023-40923-x (PMC10480200; doi:10.1038/s41598-023-40923-x)
Supplement: Supplementary file 1 — Supplementary Table 1. [file 41598_2023_40923_MOESM1_ESM.docx]

**Supplementary Table 1**: Training of the segmentation models on videos and frames of day-5 embryos.

**A.** ICM model data counts:

|  | **Videos** | **Frames** |
| --- | --- | --- |
| **Train** | 811 | 39,474 |
| **Validation** | 101 | 4,182 |
| **Test** | 102 | 5,043 |

**B.** TE diameter model data counts:

|  | **Videos** | **Frames** |
| --- | --- | --- |
| **Train** | 695 | 38,893 |
| **Validation** | 1 | 500 |
| **Test** | 32 | 5,774 |

ICM = inner cell mass, TE = trophectoderm.
